# Inhibitor Discovery of Full-Length New Delhi Metallo-β-Lactamase-1 (NDM-1)

**DOI:** 10.1371/journal.pone.0062955

**Published:** 2013-05-13

**Authors:** Bingzheng Shen, Yan Yu, Hui Chen, Xin Cao, Xingzhen Lao, Yongliang Fang, Yun Shi, Jiao Chen, Heng Zheng

**Affiliations:** 1 State Key Laboratory of Soil and Sustainable Agriculture, Institute of Soil Science, Chinese Academy of Sciences, Nanjing, Jiangsu, China; 2 Department of Pharmaceutical Bioinformatics, China Pharmaceutical University, Nanjing, Jiangsu, China; 3 Department of Gastroenterology and Hepatology, Tongji Hospital, Tongji Medical College, Huazhong University of Science and Technology, Wuhan, Hubei, China; 4 State Key Laboratory of Bio-organic and Natural Products Chemistry, Shanghai Institute of Organic Chemistry, Chinese Academy of Sciences, Shanghai, China; 5 Department of Pharmacy, Renmin Hospital of Wuhan University, Wuhan, Hubei, China; University of Westminster, United Kingdom

## Abstract

New Delhi metallo-β-lactmase-1 (NDM-1) has recently attracted extensive attention for its biological activities to catalyze the hydrolysis of almost all of β-lactam antibiotics. To study the catalytic property of NDM-1, the steady-kinetic parameters of NDM-1 toward several kinds of β-lactam antibiotics have been detected. It could effectively hydrolyze most β-lactams (*k*_cat_/*K*_m_ ratios between 0.03 to 1.28 µmol^−1^.s^−1^), except aztreonam. We also found that thiophene-carboxylic acid derivatives could inhibit NDM-1 and have shown synergistic antibacterial activity in combination with meropenem. Flexible docking and quantum mechanics (QM) study revealed electrostatic interactions between the sulfur atom of thiophene-carboxylic acid derivatives and the zinc ion of NDM-1, along with hydrogen bond between inhibitor and His189 of NDM-1. The interaction models proposed here can be used in rational design of NDM-1 inhibitors.

## Introduction

The inappropriate use and/or abuse of β-lactam antibiotics cause an increasing threaten of multi drug-resistant bacteria to public health. One of the main causes of antibiotic resistant is via the expression of β-lactamase. Two different types of β-lactamases have been discovered in clinical bacteria: the serine-β-lactamases and the metallo-β-lactamases (MβLs). MβLs require one or two zinc ions for their hydrolysis activity [1]. According to the known sequences, MβLs have been classified into three subclasses B1, B2, and B3 [2], [3].

In 2009, New Delhi metallo-β-lactmase-1 (NDM-1) was originally reported in *Klebsiella pneumoniae* from India, which belongs to the subclass B1 MβLs superfamily [4]. To date, the emergence of a large number bacteria containing *bla*_NDM-1_ gene has been reported in many other countries [5], [6]. The most troubling aspect is that these bacteria are highly resistant to almost all β-lactam antibiotics [7]–[10].

NDM–1 is a single-chain protein, which N-terminal has a putative signal peptide domain of 18 amino acids, and the core region of the enzyme composed of 270 amino acids. The crystal structures of NDM-1 [11]–[13] reveal some characteristics of this enzyme. It contains two zinc ions in the active site, near the bottom of substrate binding pocket [11]. The expanded volume of the active site and the flexible loops covering the binding pocket may explain the observed extended spectrum β–lactamase (ESBL) activity and catalytic efficiency [11]–[13].

The lack of efficient drugs against *bla*_NDM-1_-carrying strain requires continuous research effort to solve this trouble. An efficient way is to discover NDM-1 inhibitors, which can protect β-lactam antibiotics from the hydrolysis effect of NDM-1, thus recovering there antibacterial potency. Herein, we investigated the kinetic parameters of NDM-1 for the hydrolysis of nine β-lactam antibiotics. Based on biological activity determination and molecular simulation on inhibitory mechanism, thiophene-carboxylic acid derivatives were discovered to be inhibitors of NDM-1, and the inhibitory activity was proven by *in vitro* enzyme inhibition and experiment. Synergistic effect of these inhibitors in combination with meropenem against *Escherichia coli* expressing NDM-1 was also proven by determining minimal inhibitory concentration (MIC) values. The proposed model shows the detail of hydrogen bond and electrostatic interaction between compound and target, which will help us design novel and more effective inhibitors against NDM-1.

## Materials and Methods

### Construction of over-expression plasmids for soluble His_6_ tag -NDM-1 fusion proteins

The *bla*_NDM-1_ gene produced by strains of *E. coli* was chemically synthesized (Gen Bank ID: **HQ162469**) [14]. It was amplified by Polymerase Chain Reaction (PCR) with primers NDM-1-Fwd (5′-CCGGAATTCATGGAATTGC CCAATATTATGCACC-3′), which included an EcoRI restriction site (underlined) at the 5′-end of the gene, and NDM-1-Rev (5′-CCCCCAAGCTTTCAGCGCAGCTTGTCG GCCATGC-3′), which included a HindIII restriction site (underlined) after the *bla*_NDM-1_ stop codon. Amplification was carried out in 50 µL using 50 pmol of each primer and the PCR system (Takara, Osaka, Japan), under the conditions recommended by the manufacturer, and the following cycling parameters: initial denaturation at 94°C for 3 min; denaturation at 94°C for 0.5 min, annealing at 53°C for 1 min, extension at 72°C for 1 min, repeated for 30 cycles; final extension step at 72°C for 2 min. Plasmid pET28a-NDM-1 (50 ng) was used as template. The resulting PCR product was purified using a Gel/PCR Extraction kit (Biomiga, San Diego, CA). Both the PCR product and the T7-based expression vector pET30a(+) (Novagen, Madison, Wisconsin) were digested with EcoRI and HindIII for 4 h at 37°C, purified using a Biomiga kit, and ligated together to form pET30a-NDM-1. A 2 µL aliquot of the ligation mixture was used to transform 100 µL of competent *E. coli* BL21(DE3) cells (Novagen, Madison, Wisconsin), and transformants were selected for growth on solid Luria-Bertani (LB) agar plates containing kanamycin (30 µg/ml), ampicillin (50 µg/ml) and 50 µM Zn(NO_3_)_2_. Plasmid DNA purified from a single colony was sequenced and confirmed for accuracy of the NDM-1 coding sequence.

### Over-expression and purification of NDM-1 soluble proteins

The NDM-1 was produced by E. coli BL21 (DE3) carrying pET30a-NDM-1 in LB medium. Kanamycin (50 mg/ml) was used as the selecting agent during the growth of the bacteria. The inoculum was grown at 37°C until the culture reached an optical density (OD_600 nm_) of 0.8–1.0. Protein production was induced by adding isopropyl-β-D-thiogalactopyranoside (IPTG) to a final concentration of 0.1 mM. The cultures were further incubated overnight at 18°C for 14 h. The cells were harvested by centrifugation at 10000×g for 15 min at 4°C and suspended in 30 mM phosphate-buffered saline (PBS) buffer, pH 7.3. After sonication treatment, the mixture were centrifugated at 10000×g for 20 min at 4°C. The supernatant was loaded onto a Ni-NTA column (Qiagen, San Diego, CA), equilibrated with 30 mM Tris-HCl buffer (pH 7.3, containing 0.5 M NaCl and 20 mM imidazole), then the column was washed extensively with the equilibration buffer. The column was then washed with 30 mM Tris-HCl buffer (pH 7.3, containing 0.5 M NaCl and 40 mM imidazole) before elution with 30 mM Tris–HCl buffer (pH 7.3, containing 0.5 M NaCl and 100 mM imidazole). The His_6_ tag was removed by digestion with enterokinase (BBI, Ottawa, Canada) overnight at 25°C under the standard conditions of product manual. An additional step of Ni-NTA affinity chromatography was performed to remove the protease, uncut protein and affinity tag. The NDM-1 soluble protein was dialyzed against 2 L of 20 mM HEPES (Sangon, Shanghai, China) buffer (pH 6.8) overnight at 4°C and supplemented with 100 µM Zn(NO_3_)_2_. These enzymes transferred into dialysis tubing (molecular weight cutoff of 8000-14000) (Spectrum, CA, America) and overlaid with solid PEG 20000 (Merck, Darmstadt, Germany) at 4°C overnight. As a result, the NDM-1 soluble protein was concentrated 5-fold, then flash frozen and stored at −80°C. The protein concentration in the solution was determined with a commercial kit (Biomiga, San Diego, CA), with bovine serum albumin (BSA) used as a standard.

### MS

Mass Spectrometer (MS) studies were performed with a Matrix Assisted Laser Desorption Ionization Time of Flight Mass Spectrometry (MALDI-TOF MS) (BiflexIII, Bruker, Daltonik GmbH, Bremen, Germany). ZipTip (Millipore, Billerica, MA, USA) packed with C4 resin was used to prepare the solution for MALDI-TOF MS analysis of NDM-1. 1 ml of matrix solution (containing 10 mg/ml sinapic acid (SA), 0.1% trifluoroacetic acid (TFA) and 50% acetonitrile) were used to elute the NDM-1 from ZipTips and spotted onto a MALDI-TOF MS target plate. The sample spots were crystallized by air drying. NDM-1 mass was measured using the positive-ion linear mode.

### Determining the steady-state kinetic constants for various substrates

Benzylpenicillin (Sangon, Shanghai, China), ampicillin (Sangon, Shanghai, China), cefuroxime (Sigma, St. Louis, USA), ceftazidime (TCI, Shanghai, China), ceftizoxime (TCI, Shanghai, China), cefpiramide (Shandong Lukang, Shandong, China), imipenem (Shenzhen Haibin, Guangdong, China), meropenem (Zhejiang Hisun, Zhejiang, China) and aztreonam (Hunan Kelun, Hunan, China) were used as substrates for determining the steady-state kinetic constants.

Hydrolysis of the antibiotics by NDM-1 was followed by monitoring the variation in the absorbance of the β-lactam solution in 20 mM HEPES buffer, pH 6.8, 0.25 M NaCl, 2 mM 1,4-Dithio-DL-threitol (DTT) (Sangon, Shanghai, China), 100 µM Zn(NO_3_)_2_. All the measurements were made on a UV−1800 spectrophotometer (Mapada, Shanghai, China). The reactions were performed in a total volume of 100 µl at 30°C. BSA (20 µg/ml) was added to diluted solutions of β-lactamase in order to prevent enzyme denaturation. At the low concentration (20 µg/ml) used in the experiment, BSA showed no detectable interaction with the product of various substrates hydrolysis. The extinction coefficients and wavelengths used in the assays were those described by Laraki et al [15] and Thomas et al [16]. The steady-state kinetic parameters (*K*_m_ and *k*_cat_) were determined more than three times by fitting the concentration dependence of initial rate measurements to the Michaelis−Menten equation using GraphPad Prism (GraphPad Software, La Jolla, America).

### Determining the half-maximum inhibitory concentration (IC_50_) for inhibitors

The methods for synthesis of thiophene-carboxylic acid derivatives, 3-(benzyloxy)thiophene-2,5- dicarboxylic acid dimethyl ester (compound 1), 3,4-bis(benzyloxy)thiophene-2,5-dicarboxylic acid dimethyl ester (compound 2), 3,4-bis((1H-tetrazol-5-yl)methoxy)thiophene -2,5-dicarboxylic acid dimethyl ester (compound 3) and 2-(2,5-bis((1H-tetrazol-5-yl)methyl)thiophen-3-yl)malonic acid (compound 4), were reported previously [17]. Other compound, 3-thiophenemalonic acid (Shanghai Record, Shanghai, China) (compound 5), was obtained by commercial purchase. For determination of the IC_50_ of these compounds, the enzyme was pre-incubated with the desired compound for 20 min at 30°C prior to the initiation of the assay by the addition of the substrate. The compounds to be tested were dissolved in DMSO prior to incubation with the enzyme, with the final concentration of DMSO in the assay not exceeding 0.5% (vol/vol). At concentrations below 0.5%, no effect of DMSO on the enzyme activity was observed. IC_50_s were deduced from a plot of percent loss of activity versus inhibitor concentration. Meropenem (100 µM) was used as report substrate [18]. The precision of these determinations was within the range of precision 10%.

### Determining the antibacterial synergy data

*E. coli* BL21 (DE3)/pET30a-NDM-1, which is similar to clinical NDM-1-carrying isolates and safer than clinical isolates, was used to evaluate the potential ability of NDM-1 inhibitors to reverse NDM-1-mediated carbapenem resistance in bacteria. *E. coli* BL21 (DE3) was used as a control in the experiment [19]. *E. coli* BL21 (DE3)/pET30a-NDM-1 was grown overnight in MH broth media (Tianhe Microbial Agents, Hangzhou, China) containing kanamycin (30 µg/ml) at 37°C. Determination of antibacterial synergy was completed using a twofold serial dilution method, as recommended by CLSI guidelines [20]. Various concentrations of inhibitors and meropenem were added to 80 µl of a 1∶20 dilution of the overnight culture, resulting in a final volume of 100 µl. The 96-well microplates were incubated at 37°C with continuous shaking for 18 h and a reading at 630 nm was taken to determine minimum inhibitory concentrations (MICs) [21], [22]. The inhibitors were tested without meropenem at various concentrations with *E. coli* BL21 (DE3)/pET30a-NDM-1 to confirm whether the inhibitors had antibacterial activity. Each concentration reading was repeated three times. All of the above experiments were repeated at least twice.

### Molecular modeling study for interaction between inhibitor and NDM-1

Molecular docking is a molecular simulation technique, which is widely used to research the interaction between the ligand and target. Considering the flexibility of the active site in NDM-1 [11]–, flexible docking [23] implemented in Discovery Studio version2.5 (Accelrys Inc., San Diego, CA) was performed on studying the binding model. The key amino acids in active site were treated as flexible residue, which were allowed to change their conformations during docking, resulting the receptor to adapt to different ligands in an induced-fit model [24]. The crystal structure of NDM-1 in complex with hydrolyzed ampicillin was obtained from protein data bank (PDB) for flexible docking simulations (PDB ID: 3Q6X) [11]. The hydrolyzed ampicillin structure was deleted from the complex structure. 10Å from the two zincs was defined as active site covering the original area of hydrolyzed ampicillin. Water molecules in the active site were retained, for they may formed water-bridge with two zinc ions and may take part in catalyzing the hydrolysis reaction as demonstrated in many crystal structures [11], [12]. The zinc ions binding residues, His189, His120, His122, Asp124, Cys208 and His250 [11], [25], as key amino acids were also treated as flexible residues, which used for creating protein flexible conformations in the process of flexible docking. Accelrys CHARMm forcefield [26] was used throughout the simulation. All other parameters were set to default values.

To calculate single point energy, a hybrid of quantum mechanics and molecular mechanics (QM/MM) method [27] was used after the flexible-docking. The QM/MM method is necessary for NDM-1 because it can provide an accurate description of the two zinc ions−ligand−enzyme interactions, while the current force field approach failed to achieve such precision [16]. In this method, the research system was divided into a QM region and an MM region. The QM region was treated by quantum mechanics DMol^3^ [28], [29] containing His189, His120, His122, Asp124, Cys208, His250, two zinc ions and the water molecules in the active site. Becke exchange plus Lee-Yang-Parr correlation (BLYP) function [30]–[32] was used on atomic in QM region, which other parameters were default values. Other atoms were treated as the MM region by classical molecular mechanics (CHARMm).

## Results

### Over-expression and purification of NDM-1

**Figure 1:**
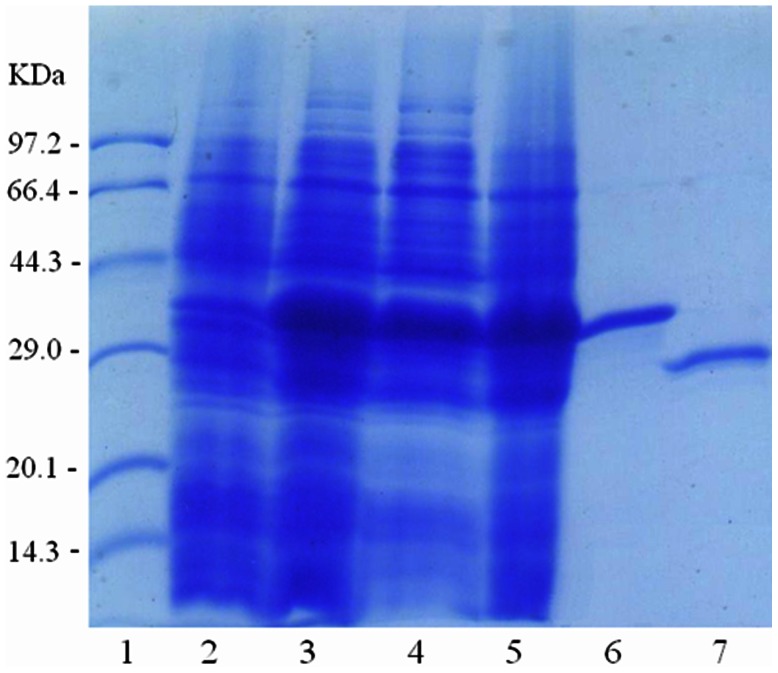
SDS-PAGE (5 to 12%) gels of NDM-1 purification. Lane 1, Takara protein molecular weight marker; lane 2, boiled cell fraction of *E. coli.*BL21(DE3) containing pET30a-NDM-1 before induction; lane 3, boiled cell fraction of *E. coli.* BL21(DE3) containing pET30a-NDM-1 after a 14 h induction with 0.1 mM IPTG at 18°C; lane 4, crude protein after ultrasonication and centrifugation in supernatant solution; lane 5, crude protein after ultrasonication and centrifugation in precipitation; lane 6, His_6_ tag-NDM-1 after Ni-NTA affinity chromatography; lane 7, full-length NDM-1 after removing the protease, uncut protein and affinity tag.

The *bla*_NDM-1_ gene was cloned in the plasmid pET30a(+), controlled by T7 promoter, and induced by 0.1 mM IPTG. The over-expression system we used was temperature-dependent on protein solubility. Protein expression at 37°C for 4 h resulted in production of an insoluble NDM-1 protein that matched the molecular weight of His_6_ tag-NDM-1, as determined by sodium dodecyl sulfate-polyacrylamide gel electrophoresis (SDS-PAGE). At low temperature condition, after induction at 18°C for 14 h, approximate half of the over-expression His_6_ tag-NDM-1 protein was soluble. Under these conditions mentioned above, 15 mg of purified His_6_ tag-NDM-1 could be isolated from 1 L of growth medium utilizing Ni-NTA affinity column. The His_6_ tag-NDM-1 was isolated to 95% purity as indicated by the SDS-PAGE. After removing the His_6_ tag, the overall yield of recombinant NDM-1 was 5 mg/L of culture. The full-length NDM-1 enzyme was isolated to 95% purity as indicated by the SDS-PAGE (Fig. 1).

### MALDI-TOF MS

MALDI-TOF MS demonstrated that recombinant His_6_ tag-NDM-1 exhibited an m/z of 34232, which was in agreement with the molecular weight deduced from amino acid sequence of 34208 m/z.

After His_6_ tag-NDM-1 was digested with enterokinase and the His_6_ tag was removed by Ni-NTA affinity chromatography, the mass spectrometry of full-length NDM-1 yielded an m/z of 29407, which was consistent with the calculated molecular mass of the mature protein of 29422 m/z.

### Steady state rate constants of full-length NDM-1 for various substrates

Steady state kinetics parameters determined using full-length NDM-1 and nine kinds of β-lactam antibiotics are summarized in Table 1.

**Table 1:** Steady state kinetic parameters of full-length NDM-1with nine kinds of β-lactam antibiotics.

Substrate	Concn range (µM)	Enzyme (µM)	*K*_m_ (µM)	*V*_max_(µMs^-1^)	*k*_cat_ (s^−1^)	*k*_cat_/*K*_m_ (µM^−1^s^−1^)
Penicillins						
Benzylpenicillin	100–2,000	0.5–1.5	13±1	8.35±0.79	16.7	1.28
Ampicillin	200–2,000	0.5–1.5	18±1	5.7±0.44	11.4	0.63
Cephalosporins						
Cefuroxime	20–200	0.5–1.5	12±1	1.3±0.11	2.6	0.22
Ceftazidime	10–150	0.5–1.5	129±9	1.8±0.08	3.6	0.03
Ceftizoxime	20–250	0.5–1.5	29±2	1.5±0.09	3.0	0.10
Cefpiramide	10–200	0.5–1.5	39±3	0.7±0.05	1.4	0.04
Carbapenems						
Imipenem	20–1,000	0.2–1.5	127±11	5.4±0.35	10.8	0.09
Meropenem	10–300	0.2–1.5	68±6	2.0±0.15	4.0	0.06
Monobactam						
Aztreonam	500–1,500	0.5–1.5	ND a	ND	ND	

a ND, not determined.

The kinetic parameters presented that NDM–1 is an extended spectrum β-lactamase. It could hydrolyze nearly all classes of β-lactams antibiotics, with the exception of aztreonam. The *K*_m_ values displayed that NDM-1 has higher affinity to most penicillin and cephalosporin antibiotics with the exception of ceftazidime, while relatively lower affinity with carbapenems. NDM-1 exhibited *k*_cat_ values greater than 1s^−1^ for all penicillins, cephalosporins and carbapenems. Among all substrates, the relatively higher *k*_cat_ values (more than 10s^−1^) were shown by benzylpenicillin, ampicillin, and imipenem. The highest *k*_cat_/*K*_m_ ratio for all substrates was benzylpenicillin (1.28 µM^−1^s^−1^), deduced from kinetic parameters. In the case of the penicillin class of β−lactam antibiotics, NDM-1 displayed less *K*_m_ values for benzylpenicillin (13 µM) than ampicillin (18 µM), which illustrated benzylpenicillin bound with NDM-1 more tightly. The *k*_cat_ value for benzylpenicillin (16.7s^−1^) was also greater than ampicillin (11.4s^−1^). Due to the greater value of *k*_cat_/*K*_m_ ratio, NDM-1 presented higher catalytic activity for benzylpenicillin (1.28 µM^−1^s^−1^) than ampicillin (0.63 µM^−1^s^−1^). With respect to the cephalosporin class of β-lactam antibiotics, these four substrates displayed *K*_m_ values ranging from 12 to 129 µM. In particular, ceftazidime displayed greatest *K*_m_ value (129 µM), which indicated binging most weakly to NDM-1 in all tested cephalosporin class antibiotics, and exhibited highest *k*_cat_ value (3.6s^−1^) in this class of antibiotics. Cefuroxime presented the lowest *K*_m_ value (12 µM) and the highest value of *k*_cat_/*K*_m_ ratio (0.22 µM^−1^s^−1^), which revealed cefuroxime was the best substrate for these four substrates. With reference to carbapenem class antibiotics, *K*_m_ value for imipenem (127 µM) was greater than meropenem (68 µM), while *k*_cat_ value for imipenem (10.8s^−1^) was also greater than meropenem (4.0s^−1^). The trend of binding affinity as reflected in *K*_m_ values and speed of catalysis as reflected in *k*_cat_ values somewhat offset each other, leading that NDM-1 presented similar catalytic activity as reflected in values of *k*_cat_/*K*_m_ ratio for imipenem (0.09 µM^−1^s^−1^) and meropenem (0.06 µM^−1^s^−1^). Under the experimental conditions employed, NDM-1 hydrolyzed all the tested compounds except aztreonam. In spite of the fluctuation in the absorbance of this substrate have been observed, kinetic constants could not be calculated for the monobactam aztreonam.

### Inhibitory activities of compounds

**Figure 2:**
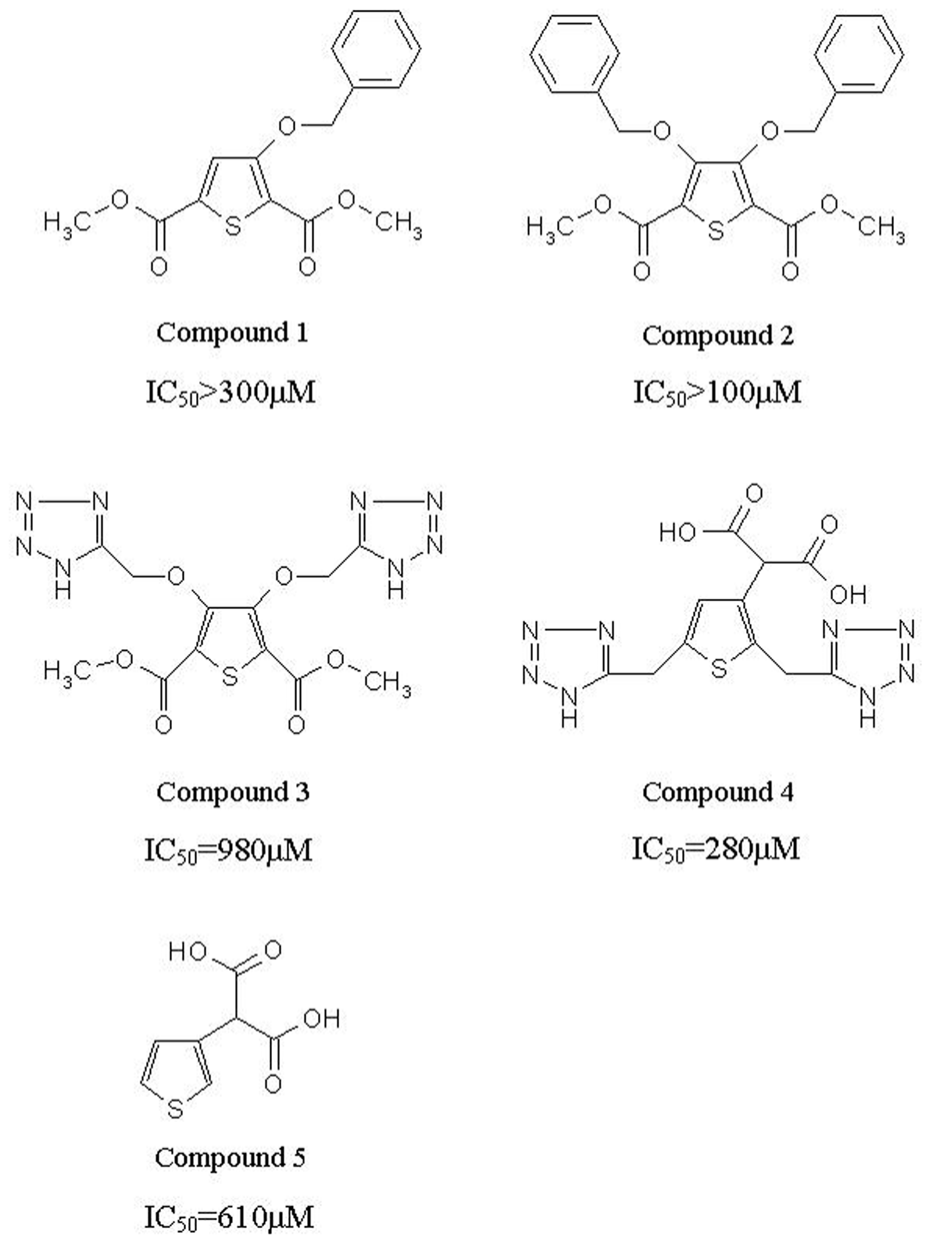
Molecular structures and IC_50_ values of inhibitors against NDM-1. Effects of inhibitors against full-length NDM-1 activity were mediated by meropenem hydrolysis. The precision of the IC_50_ determinations was within the range of ±10%.

Inhibitory activities of all compounds were determined using meropenem as report substrate by the methods described above. The results, as shown in Fig. 2, indicated that thiophene-carboxylic acid derivatives had inhibition effect on NDM-1.

**Figure 3:**
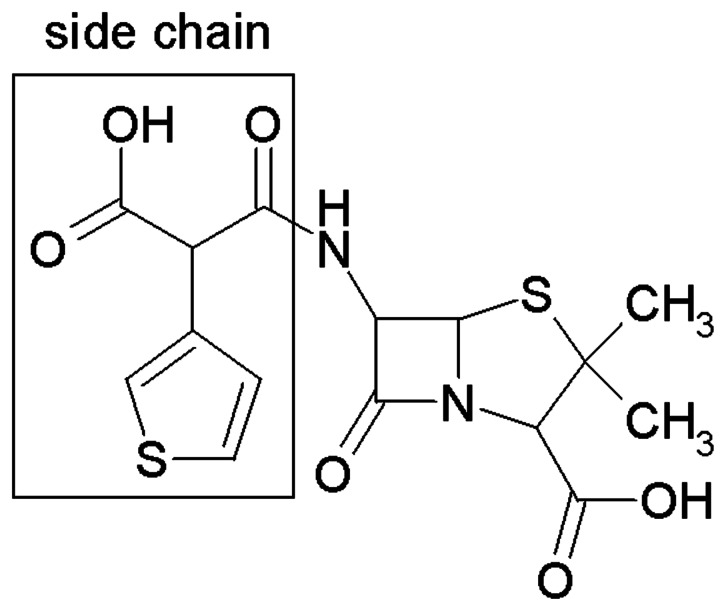
Structure of ticarcillin.

Because of containing the strong hydrophobic benzyl group in structure, compounds 1 and 2 precipitated out at high concentrations in testing buffer, the residual NDM-1 activity for different concentrations could not be obtained in most instances (compound 1 IC_50_>300 µM and compound 2 IC_50_>100 µM). Compound 3 had hydrophilic substructure of two (1H-tetrazol-5-yl)methoxy groups, leading to a better solubility. Among the all thiophene-carboxylic acid derivatives we have synthesized, compound 3 showed weak inhibitory activity against NDM-1 (IC_50_ = 980 µM). In comparison with compound 3, compound 4 with two carboxyl groups, presented stronger solubility and medium inhibitory effect on NDM-1 (IC_50_ = 280 µM). The deduction that two methoxycarbonyl groups in the structure of compound 3 were not necessary for inhibitory activity could be drawn. It was almost certain that and carboxyl groups, as well as (1H-tetrazol-5-yl)methoxy groups, played an important contribution to the inhibitory effect. It is interesting that compound 5 (3-thiophenemalonic acid), exhibited weak inhibitory ability (IC_50_ = 610 µM). This compound is the side chain of ticarcillin (Fig. 3), a clinical used drug to treat gram-negative bacteria infection, indicating that this substructure may has favorable bioavailability and low toxicity, which may serve as a lead compound for further investigation.

### Antibacterial activity of meropenem in combination with NDM-1 inhibitors

*E. coli* BL21 (DE3)/pET30a-NDM-1 presented high resistance (MIC 256 µg/ml) compared to *E. coli* BL21 (DE3) (MIC 2 µg/ml), which resulted in a 128-fold resistance to meropenem. The MICs of meropenem in combination with NDM-1 inhibitors at various concentrations against *E. coli* BL21 (DE3)/pET30a-NDM-1 were presented in Table 2.

**Table 2:** Synergistic antibacterial activity of NDM-1 inhibitors in combination with meropenem against *E. coli* BL21 (DE3)/pET30a-NDM-1.

Compounds	MICs (µg/ml) of meropenem in combination with NDM-1 inhibitors at various concentrations against *E. coli* BL21 (DE3)/pET30a-NDM-1				
	100.0 µM	50.0 µM	25.0 µM	12.5 µM	6.25 µM
Synthesis					
1	ND a	256	256	256	256
2	ND	256	256	256	256
3	32	32	128	256	256
4	16	32	64	64	128
Commercial purchase					
5	16	32	64	128	256

a ND, not determined.

Note that a meropenem concentration ranging from 17.3 to 31.7 µg/ml is clinically achievable [33].

Compounds 3, 4 and 5 showed synergistic antibacterial activity with meropenem. In these three inhibitors, compounds 4 and 5 were able to sensitize the strain to clinically achievable concentrations of meropenem at the level of 100 µM. The least potent of the inhibitors, compound 1 and 2, did not exhibited synergistic antibacterial effect with meropenem. The MIC values of meropenem in combination with various compounds at the same concentration exhibited that the different substituents of the mother nucleus had a great influence on synergistic antibacterial activity, which the (1H-tetrazol-5-yl)methoxy group was better. The benzyloxy substituents of compounds 1 and 2 led to failure in synergistic antibacterial effect.

### Flexible docking and QM/MM study of binding model and interaction between inhibitors and NDM-1

**Figure 4:**
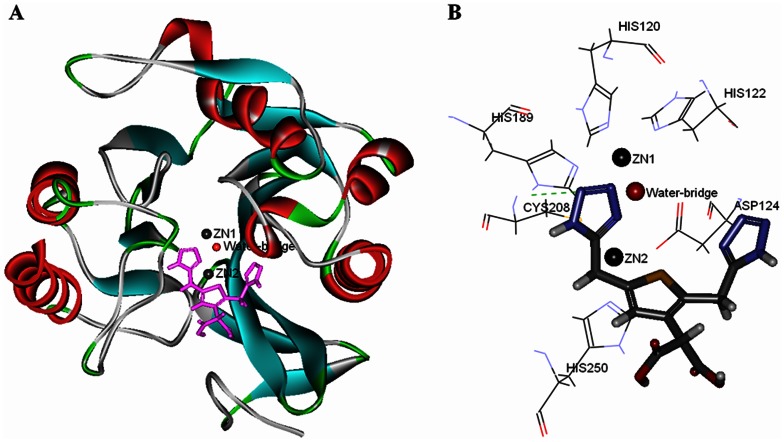
Binding model between compound 4 and NDM-1 in active site. NDM-1 and compound 4 is shown as solid ribbon model and a stick model. Water-bridge and two zinc ions are shown as spheres, respectively. Key amino acids, His189, His120, His122, Asp124, Cys208 and His250, in active site are shown as line model.

To investigate the potential binding molecular modeling of compound 4 to NDM-1, flexible docking was employed. Compound 4 was docked into the active site of NDM-1, which was located at the bottom of a shallow groove (Fig. 4A).

**Figure 5:**
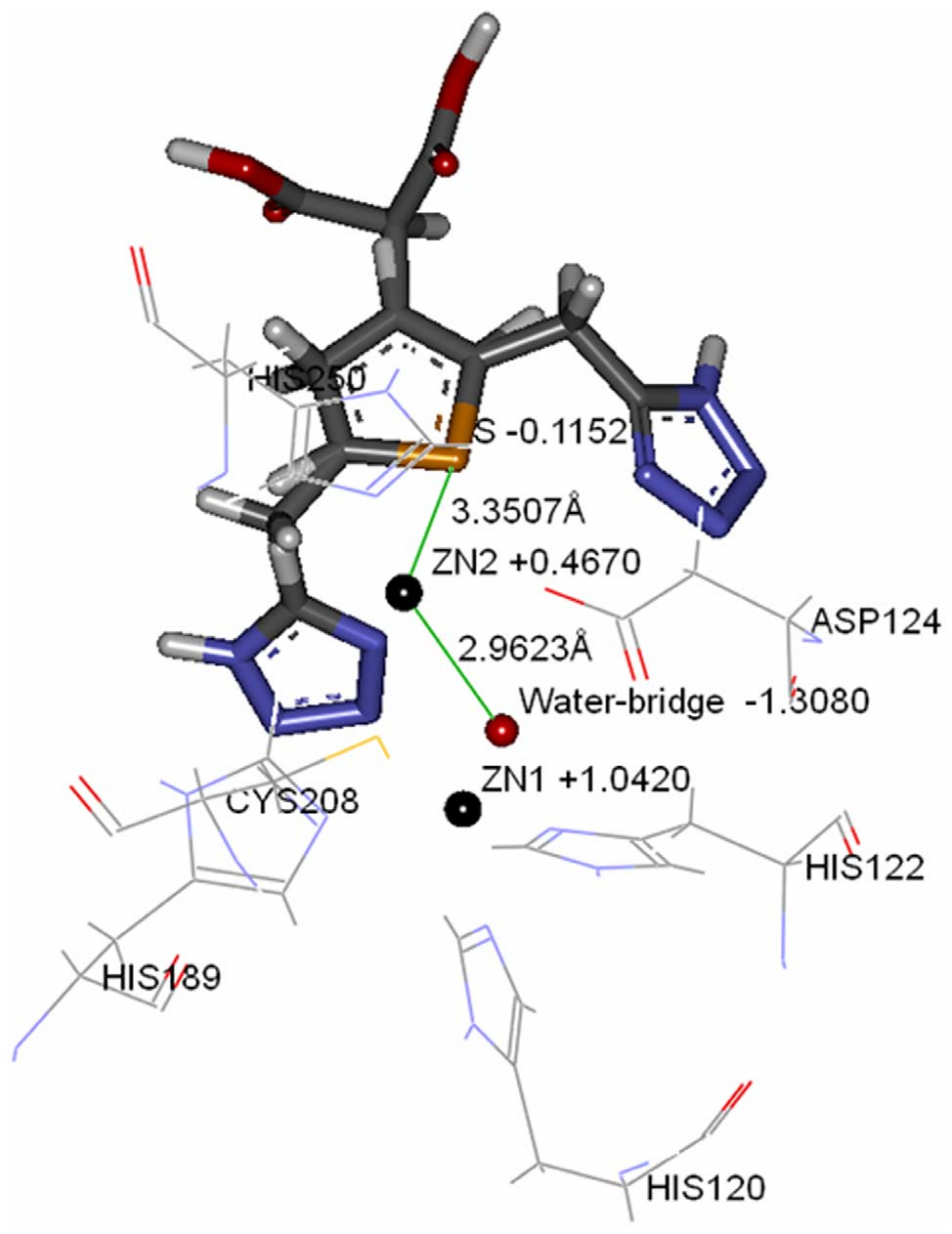
The accurate partial charge values of two zinc ions, water-bridge and sulfur atom of compound 4. Water-bridge and two zinc ions are shown as spheres. Compound 4 and key amino acids are shown as stick model and line model, respectively.

The flexible docking modeling illustrated that compound 4 have bound within the active site occupying the groove parallel to the flexible loop and close to the two zinc ions. The results also demonstrated that hydrogen atom of imidazole ring of His189 and (1H-tetrazol-5-yl)methoxy groups of compound 4 could form hydrogen bond (Fig. 4B). Apart from the interaction of hydrogen bond, the sulfur atom of the thiophene group of compound 4 might play an important role in electrostatic interactions to enhance the binding affinity of the inhibitor (Fig. 5). The sulfur atom of compound 4 was located close to zinc2 ion in the active site. The distance between them was 3.35Å, which was 0.39Å longer than the distance between oxygen atom of water-bridge and zinc2 ion (2.96Å). Encouragingly, the atomic radius of sulfur atom (1.04Å) is 0.38Å bigger than oxygen atom (0.66Å). Based on the results and data analysis, the deduction of electrostatic interactions between the sulfur atom of compound 4 and zinc2 ion was plausible. The results of QM/MM research on the water-bridge, two zinc ions, and compound 4 also demonstrated our deduction (Fig. 5). The accurate partial charge values of zinc2 ion and sulfur atom of compound 4 were +0.4670 and −0.1152, causing the electrostatic interaction between partial positive and partial negative charge.

## Discussion

Based on the knowledge of the NDM-1 crystal structure, a protein–ligand interaction model for thiophene-carboxylic acid inhibitor was proposed by QM/MM method. By virtue of most classical force fields using fixed-charge proximity to treat electrostatic interactions, the standard MM method was found to be irrational and inaccurate in our system. It is difficult to compute the polarization and charge transfer state of zinc ion [34]. QM is an accurate and efficient method to calculate partial charge and energy, but it has disadvantages in calculate huge system for its relatively low speed. To this end, a hybrid QM/MM approach was chosen for NDM-1, which proved to be an effective tool to handle, metal ion and key amino acids residues in target protein in relatively high speed and reliable accuracy. The different partial charge of two zinc ions (Zn1 +1.0420 and Zn2 +0.4670) presented in our system reflected the disparate zinc ion function in catalytic reaction mechanism, which verified the deductions proposed by Zhang et al [11] and Yang et al [35]. We also proposed here that the distance change between zinc binding residue and zinc ion may affect the charge of zinc ion, thus changing the kinetic property of the enzyme. Further studies on NDM-1 mutations are in progress, which may give deep insight on the relationship between zinc ion charge and NDM-1 catalytic property.

According to the IC_50_ values and MIC values, it was presented that the compounds, which displayed the potent inhibitory activities against NDM-1 enzyme, also had the preferable synergistic antibacterial effect of meropenem against *E. coli* BL21 (DE3)/pET30a-NDM-1. The inhibitors, thiophene-carboxylic acid derivatives (compound 4 and compound 5), might be used as a potential skeleton compound in the discovery of antibacterial agents. Most inhibitors of MβLs interact with the enzyme through weaker forms of non covalent bonds, including hydrogen bonds, van der Waals interactions, dipole–dipole interactions and hydrophobic interactions. Electrostatic interactions or ionic bonds, which have been proven unnegligible effect in ligand-protein interaction by the QM/MM molecular simulation method, can be used to guide structural modifications or design new structure inhibitors.

For the sake of increasing inhibitory activity of these compounds, in addition to electrostatic interactions, substituent group and side chain of mother nucleus of thiophene-carboxylic acid, should be paid more attention. As mentioned previously, the active site of full-length NDM-1 is composed of two zinc ions, water-bridge and six key amino acids (His189, His120, His122, Asp124, Cys208 and His250). As showed in the binding model, sulfur atoms of thiophene ring have influenced the interaction between the oxygen atom of water-bridge and two zinc ions. And yet, the side chain of thiophene-carboxylic acid have played weak interaction of side chain group with only His189 amino acid residue of six key amino acids, which is the main reason for the inhibitory activity is not very satisfactory.

The structural modification may allow substituent group and side chain to sufficiently interact with the groups of six key amino acids. If these compounds have enough bonding strength after structural modification, they can bind tightly with target and block the entry of substrate into the active site of NDM-1. Now, further chemical modifications of these compounds are underway.
